# Fetal Growth Restriction Prediction: How to Move beyond

**DOI:** 10.1155/2019/1519048

**Published:** 2019-08-21

**Authors:** Debora F. B. Leite, Jose G. Cecatti

**Affiliations:** ^1^Department of Obstetrics and Gynecology, University of Campinas, School of Medical Sciences, Campinas, Sao Paulo, Brazil; ^2^Federal University of Pernambuco, Caruaru, Pernambuco, Brazil; ^3^Clinics Hospital of the Federal University of Pernambuco, Recife, Pernambuco, Brazil

## Abstract

The actual burden and future burden of the small-for-gestational-age (SGA) babies turn their screening in pregnancy a question of major concern for clinicians and policymakers. Half of stillbirths are due to growth restriction *in utero*, and possibly, a quarter of livebirths of low- and middle-income countries are SGA. Growing body of evidence shows their higher risk of adverse outcomes at any period of life, including increased rates of neurologic delay, noncommunicable chronic diseases (central obesity and metabolic syndrome), and mortality. Although there is no consensus regarding its definition, birthweight centile threshold, or follow-up, we believe birthweight <10^th^ centile is the most suitable cutoff for clinical and epidemiological purposes. Maternal clinical factors have modest predictive accuracy; being born SGA appears to be of transgenerational heredity. Addition of ultrasound parameters improves prediction models, especially using estimated fetal weight and abdominal circumference in the 3^rd^ trimester of pregnancy. Placental growth factor levels are decreased in SGA pregnancies, and it is the most promising biomarker in differentiating angiogenesis-related SGA from other causes. Unfortunately, however, only few societies recommend universal screening. SGA evaluation is the first step of a multidimensional approach, which includes adequate management and long-term follow-up of these newborns. Apart from only meliorating perinatal outcomes, we hypothesize SGA screening is a key for socioeconomic progress.

## 1. Introduction

The intrauterine environment influence on fetus development is a well-known determinant of individual's long-term health and quality of life. From the initial description of 23 infants being born at term weighing less than 2000 g, Warkany et al. [1] introduced the idea of “intrauterine growth retardation” (IUGR). Soon, they were followed by others [2–4]. They considered IUGR “all conditions leading to a marked reduction in size during intrauterine life” [1], mainly represented by reduced birthweight. Although all of them have described pregnancies and infants with a wide variation of phenotype, with and without hypertensive syndromes or morphologic anomalies, for instance, the turning points were to consider the environment in which the fetus was developing and the placenta role in this process.

In fact, human development goes far beyond genetic inheritance. Lessons learned from pregnancies subjected to smoking [5, 6] or intermittent fasting [7], for instance, show how intrauterine growth is adjustable. Posttranslational changes in small-for-gestational-age (SGA) infants [8] reinforce the thrifty phenotype hypothesis [9]. According to Hales and Barker, the nutritional deficiency, especially regarding amino acids supply, would decrease the pancreatic beta-cells function and induce changes of the muscular, hepatic, and adipose tissue systems functioning, for example [9]. These newborns are at higher risk of neonatal morbidity and mortality [6, 10–18]; in adolescence and adulthood, they present worse neurodevelopment [19, 20] and metabolic [21, 22] and cardiovascular [23] adverse outcomes. On the contrary, the placenta—a shared organ by both the mother and the fetus—is responsible for adjusting maternal supply to the fetus demands. Since it is difficult to realize which are the normal placental functioning patterns and the optimal fetal growth, it is reasonable to use the birthweight as a measure of the intrauterine environment [24] and SGA newborns as surrogates for fetal growth restriction (FGR) [10–13].

Therefore, considering the long latency of some events, such as cognitive delays and cardiovascular diseases, SGA has impacts of public health magnitude, especially in low- and middle-income countries (LMICs) [13, 25]. In this review, we will discuss the importance of SGA screening in pregnancy and which are the best approaches and moments to perform it.

## 2. Why We Should Screen for Fetal Growth Restriction?

The identification of FGR as a distinct pathophysiological entity is merged with preterm birth history. In the first half of the 20^th^ century, gestational age at birth and birthweight concepts overlapped; the World Health Organization recommended a birth weight of 2500 g or less to characterize prematurity [26]. However, several authors and clinicians were intrigued by “pseudopremature” newborns, who would be in chronic suffering due to placental insufficiency and would benefit from earlier delivery [2–4]. Only in 1961, the terminology IUGR was first cited [1]. Apart from only birthweight (<2000 g), Warkany et al. suggested that preterm infants whose birthweight were 40% below the expected for a given gestational age should be considered IUGR. Two years later, Battaglia and Lubchenco proposed to use the birthweight as a proxy for intrauterine development [27] and this is still a common practice in the 2000s [10–13], due to difficulties in defining and measuring fetal growth [28–30].

Currently, the birthweight <10^th^ centile, either by population-based or customized charts, is the most accepted definition for SGA infants [28]. This mathematical threshold was initially chosen due to (i) the increased neonatal mortality observed in this group when compared to those born between the 10^th^ and the 90^th^ centiles and (ii) the agreement on the 10^th^ centile among studies up to the 1960s [27]. There are concerns that some of these infants are “constitutionally small,” not at higher risk of (neonatal) adverse outcomes, and lower limits for SGA, such as ≤5^th^ [31], ≤3^rd^ or even ≤2, and 3^rd^ centile [32], are considered by some researchers. However, little is still known about the long-term health endpoints of the “constitutionally small” newborns. Therefore, the 10^th^ centile seems the most suitable cutoff for epidemiological and clinical purposes, and it is the adopted threshold in this review.

The SGA prevalence varies according to the reference standards applied; it tends to be higher with customized curves [11, 12, 14]. Using population-based charts, live births between 19.3% [13] and 27% [25] in LMIC could have been classified as SGA in 2000s. A majority of them were term-SGA (98% and 95.6%, respectively). This turns SGA the most important pregnancy-related syndrome since other pathological conditions, such as pregnancy hypertension and preterm birth, have markedly lower prevalence [11, 12, 14]. It is interesting to note, however, that these “great obstetrical syndromes” may share pathophysiological pathways [33], and it is possible that SGA may represent an underlying condition for the other ones.

The “great obstetrical syndromes” are related to defective deep placentation [33], and studies on placental biomarkers point in this direction [34, 35]. Not surprisingly, pathological placental findings have been related to SGA pregnancies, especially vascular malperfusions lesions, infarction, and chronic villitis of unknown etiology [36–39]. Vascular-mediated changes (e.g., decidual vasculopathy and single or multiple infarctions) usually coexist with Doppler (uterine (UtA), umbilical (UA), or middle cerebral (MCA) arteries) [37, 39] or biochemical abnormalities (such as low levels of placental growth factor (PlGF) [35] or alpha-fetoprotein (AFP): pregnancy-associated plasma protein-A (PAPP-A) ratio >10 [34]). These pathological and functional observations are similar to those found in pregnancies affected by hypertensive disorders, preterm deliveries, and stillbirth [40–44], then possibly reflecting an elementary chronic hypoxia mediating these outcomes.

Additionally, some clinical risk factors are similar between the “great obstetrical syndromes.” Multiple pregnancies and maternal chronic conditions, such as previous hypertension, systemic lupus erythematosus, and diabetes mellitus, are all associated with them [45–47]. Nulliparity [11, 14, 48], shorter height [11, 14, 48], lower pre-pregnancy weight [11, 14, 48] or body mass index [11, 14], previous history of SGA [6, 11, 48], smoking [5, 6, 11, 32, 48], and being born SGA [49] are frequently related to SGA pregnancies. Maternal age shows conflicting results, as well as ethnicity [11, 14], socioeconomic, and marital status [45], which may explain how maternal culture background and environment influence SGA patterns in a given population.

Regarding the outcomes of SGA newborns, extensive investigation has been performed on immediate [10–14, 16, 18, 32, 50] and long-term endpoints [19–23, 51, 52], demonstrating worse health performance at any period of life. Not surprisingly, the leading countries in absolute numbers of fetal and neonatal deaths [53] are the same as SGA [25]: India, Pakistan, and Nigeria. Indeed, growth restriction can account for up to half of the fetal deaths of unknown causes [54], being about 6-fold higher than the chance of stillbirth at term (relative risk, RR, 6.0; 95% CI, 3.1–11.5) [11], or when the birthweight is <5^th^ percentile (compared to the 10–90^th^ centiles) [17]. Besides perinatal death [6, 11, 12, 15–18, 55], preterm birth [6, 11, 14], and other short-term adverse events are described for SGA infants (Table 1); the adjusted odds ratio (aOR) for composite neonatal morbidity can be as high as 3.22 (95% CI, 3.07–3.39) [12]. Interestingly, SGA suspicion in pregnancy is associated with better neonatal outcomes [18, 57], which turns SGA screening a cornerstone strategy for reducing antepartum fetal loss [13, 58] and meliorating neonatal morbidity ratios.

Unfortunately, the higher risk of mortality goes beyond the neonatal period. Data from Sweden show a hazard ratio (HR) of 1.37 (95% CI 1.28–1.47) of death up to 18 years old, which increased to 2.61 (95% CI 2.19–3.10) for those neonates born <3^rd^ centile [59]. Additionally, growth restriction is associated with a lower Bailey score, especially in communication skills domain [19], sleep disorders [52], and hyperactivity [56]. If SGA fetuses experience any degree of brain-sparing effect, the delayed motor skills and cognitive development are even more pronounced [19, 51]. Regarding metabolic repercussions, insulin and insulin resistance index (HOMA IR) are higher in SGA children at 6–8 years old and those born <3^rd^ centile also have higher levels of leptin [22].

Evidence from adults exposed to famine *in utero* shows increased odds for metabolic syndrome [21] and obesity [60] in SGA newborns, perhaps in a sex-specific manner, depending on childhood nutritional parameters (especially weight gain velocity). Proportionate biometric measurements at birth were the initial observations of Barker et al. who related the ponderal index, head circumference, and birthweight <2495 g to cardiovascular mortality [23]. Although maternal undernourishment is not synonymous of SGA infant and considering that the birthweight approach has changed over time, these findings mean that adequate fetal development is the standpoint for long-term health. There is greater visceral fat thickness (in women) [61], higher fat-free soft tissue mass [62], and increased trunk and abdominal fat mass proportion (of both sexes) [63] in adults born SGA. These epidemiological data ground current theories of epigenetic modifications in SGA infants, leading to enriched (i.e., with increased DNA methylation) pathways involved with fat, sugar, and protein metabolism [8].

Therefore, timely recognition of SGA—still in pregnancy—is a real concern for obstetricians, perinatologists, health workers, and policymakers. Unfortunately, only a small proportion of SGA babies are suspected before birth [18, 57], leading to a lack of appropriate short- and long-term follow-up of these newborns. SGA suspicion will provide adequate management of the mother and fetus/newborn, including referencing to a specialized facility for antenatal care and delivery and individualized follow-up in childhood, adolescence, and adulthood.

## 3. When and How We Should Screen for Fetal Growth Restriction?

### 3.1. Clinical Factors

Clinical risk assessment is the first approach to antenatal care. A detailed maternal history at booking can identify several risk factors and guide referencing to tertiary care facilities.

Single maternal clinical factors demonstrate poor prediction accuracy (Table 2), and, as a result, are generally considered in a multidimensional model. Smoking, although less prevalent in the early years of the 21^st^ century, still demonstrates effects on fetal growth [5, 6, 48] and is the most common maternal variable to compose a prediction model. Lower maternal stature and weight appear associated with SGA in some studies [11, 14, 48] but showed only 43% and 73% of sensitivity, respectively [64]. Body mass index (BMI) and maternal weight gain throughout pregnancy demonstrate an area under the (AUC) receiver operating characteristic (ROC) curve of 0.56 and 0.60, respectively [64]. The performance of symphysial-fundal height (SFH) measurement in predicting SGA newborns increases with gestational age [68], but it is not different to Leopold's maneuvers (RR1. 32, 95% CI 0.92–1.90) [69]. However, since it is inexpensive and already part of the routine obstetrical examination, Cochrane reviewers advise its use and health professionals should associate it with some other technique or evaluation of fetal growth.

Other maternal factors have been combined differently, evidencing how SGA syndrome can be heterogeneous in distinct settings. In a multicenter international nulliparous cohort, a family history of coronary heart disease, maternal birthweight <3000 g, infertility, college student, smoking at the 2^nd^ trimester, proteinuria, daily vigorous exercise, and diastolic blood pressure ≥80 mmHg, combined with the protective factors rising random glucose, recreational walking (≥4x/week), and *Rhesus* negative blood group, provided an AUC of 0.63 [6]. This same AUC (0.66, 95% CI 0.61–0.70) was achieved by combining maternal age and height, smoking, previous SGA infant, and chronic hypertension in Spain [70]. In the United Kingdom, a logistic regression model included maternal height, weight, parity, ethnic background, smoking, and previous history of preeclampsia or SGA [71]. In this model, maternal factors evaluation between 35 and 37 w have had similar AUC for delivery within two weeks (0.744; 95% CI 0.731–0.756) and term delivery (0.712; 95% CI 0.700–0.725) for SGA without preeclampsia.

### 3.2. Ultrasound Scans

Adding ultrasound scan (US) parameters to maternal clinical factors improves the performance of prediction models, although not consistently [6, 71]. Crown-rump length (CRL) [43]; nuchal translucency (NT) [43]; head circumference (HC) [6]; abdominal circumference (AC) [6, 72]; AC growth velocity (ACGV) [72, 73]; femur length (FL) [74]; estimated fetal weight (EFW) [32, 44, 71, 73, 75, 76]; uterine arteries pulsatility index (UtA-PI) or resistance (UtA-RI) index, or notches [6, 32, 71, 76]; umbilical artery PI (UA-PI); middle cerebral artery PI (MCA-PI); cerebral-placental ratio (CPR: MCA-PI/UA-PI) [32, 66, 76]; and umbilical vein blood flow (UVBF) [32] were studied for SGA prediction. Except for NT, the lower the fetal biometry, the higher the odds for SGA; in general, there is a trend towards better US predictive accuracy for lower birthweight centiles (especially <3^rd^) [67, 76]. Unfortunately, participants' selection criteria, study protocol of follow-up, and outcome measures differ between studies, precluding interpretation and evaluation of US in clinical practice [67]. In Table 2, predictive accuracy measures of EFW and AC are shown.

In the 1^st^ trimester, decreased values of NT were associated to lesser odds for SGA (OR 0.79; 95% CI 0.70–0.89), but the CRL has shown no relationship (OR 0.99, 95% CI 0.99–1.00) [43]. In the 2^nd^ trimester, McCowan et al. [6] demonstrated that only a limited increase in AUC (from 0.66 to 0.73) was observed with the addition of 20 w US data to maternal data: HC *z*-score <10^th^ centile, AC *z*-score <10^th^ centile, and UtA-RI ≥ 0.05. The higher the UtA-RI, the higher the OR for SGA, reaching 4.56 (95% CI 2.45 to 8.48) when 0.8–1.0. At 35–37 w, Fadigas et al. [71] have combined maternal variables with EFW *z*-score, which improved AUC from 0.81 (95% CI 0.802–0.824) to 0.98 (95% CI 0.98–0.98) for delivering an SGA infant <3^rd^ centile in less than two weeks. In this cohort, adding mean arterial pressure and UtA-PI has not improved the prediction performance (AUC 0.98; 95% CI 0.98–0.99).

Interestingly, a single measurement is better than longitudinal follow-up [32, 67, 72, 73, 75]. In the 2nd trimester, the femur length <5^th^ centile is associated with increased odds for IUGR or SGA (3.24, 95% CI 2.34–4.48) [74]. The performance of EFW <10^th^ centile at 35–37 w in predicting delivery within two weeks is better for SGA <3^rd^ (83%; 95% CI 80–86) than for SGA <10^th^ (69%; 95% CI 67–71) [76]. Although EFW <10^th^ centile is related to a higher risk of any adverse perinatal outcomes [77], it demonstrates poor prediction performance (26%; 95% CI 28–30; for SGA <3^rd^ delivering in 2 w) [76]. In another example, Triunfo et al. have demonstrated better prediction performance of EFW at 37 w for SGA <3^rd^ (0.85; 95% CI 0.82–0.89), when compared to the 4–10^th^ centiles (0.93; 95% CI 0.89–0.97) but reached a disappointing AUC of 0.54 (95% CI 0.48–0.61) for predicting adverse perinatal outcomes [32]. This is also true for AC cross-sectional evaluation at 32 w seemed compared to ACGV (difference from 32 w results and 2^nd^ trimester) [72]. The detection rate (DR) of SGA <10th centile was 49.1 (95% CI 44.2–52.8; false positive rate, FPR, 10%) and 81.2 (95% CI 75.3–88.1) for SGA <3^rd^ centile or suspected before birth by abnormal Doppler results. This finding partially contradicts the Pregnancy Outcome Prediction (POP) Study, which found a relative risk of 17.6 (95% CI 9.2–34.0) for delivering an SGA infant when both EFW and ACGV (between 28 and 36 w) were <10th centile [73]. In this study, the sensitivity of EFW <10^th^ centile was higher with universal screening for SGA <10^th^ (57%) or <3^rd^ centile (77%) than with clinically oriented US evaluation (20% and 32%, respectively) [73].

More recently, magnetic resonance imaging (MRI) has been explored in maternal-fetal surveillance. Carlin et al. [78] have demonstrated no difference in EFW ≤3^rd^ or ≤5^th^ centile by US or MRI before delivery (48 h). However, DR of SGA ≤10^th^ centile was superior with MRI (100.0; 95% CI 81.5–100.0, FPR of 10%) than the US (77.8; 95% CI 52.4–93.6, FPR 10%).

### 3.3. Biomarkers

Biomarker measurements of placental functioning-related substances have had significant development in the last three decades. Many of these compounds are also involved with antenatal detection of chromosomal anomalies, or preeclampsia, such as PAPP-A, AFP, PlGF, or sFLt-1 [41] (Table 2). Studies from the mid 1980s have evaluated the human placental lactogen (hPL), when it demonstrated a diagnostic odds ratio (DOR) of 4.78 (95% CI 3.21–7.13), whereas more recent data focus on angiogenic biomarkers [44].

The AFP: PAPP-A ratio >10 at 12 w of pregnancy provided a risk ratio of 3.74 (95% CI 2.3–6.09) for SGA <3^rd^ centile [34]. In early 2^nd^ trimester (15 w), serum levels of PAPP-A, PlGF, and insulin are significantly lower in SGA pregnancies [79], while increased plasma levels of vascular growth factor (VEGF) between 34 and 37 weeks were related to a lower chance of restricted fetuses (OR 0, 8; 95% CI 0, 71–0, 92) [80]. Conversely, a model built by EFW, UtA-PI, and PlGF at 35–37w has provided an AUC 0.883 (95% CI 0.867–0.899) [81].

PlGF has consistently lower levels in SGA pregnancies, in 2^nd^ and 3^rd^ trimesters [82–84], especially for BW < 5^th^ or <10^th^ centiles. For higher sFlt-1/PlGF ratios, there is better AUC for preeclampsia-associated SGA [40, 41]. These findings point in the direction of angiogenesis-mediated pathophysiology of SGA, especially when there are Doppler abnormal parameters [37]. Unfortunately, PlGF shows poor accuracy to be implemented in clinical practice: the combined AUC was 0, 66 (95% IC 0, 44–0, 87) for FGR prediction [65]. Perhaps, this finding is due to the diverse PlGF measurements and FGR definitions used by the studies included in the systematic review, which considered either the estimated fetal weight, birth weight, or the presence of additional findings of severity (e.g., oligohydramnios).

After all, better accuracy was achieved by combining multiple maternal, ultrasonographic, and biochemical clinical factors. In an international cohort of nulliparous women [79], PlGF has had an AUC of 0.84 (95% CI 0.78–0.89) for hypertensive-SGA when combined with smoking, proteinuria, uterine artery Doppler, PAPP-A, and triglycerides. In the 2^nd^ trimester (19–24 w), PlGF and AFP, combined with maternal factors and fetal biometry, made up an AUC of 0.96 for birth below 32 weeks in SGA newborns [31].

## 4. Conclusions

Fetal growth restriction is related to adverse outcomes in the perinatal period, childhood, and adulthood; the estimated actual burden of SGA [13, 25] might be even higher in the next few years. Starting antenatal care at early pregnancy leads to adequate risk management and additional evaluation assessment, with US or biomarkers. The “inverted pyramid” of prenatal care claims attention to the early pregnancy risk evaluation [85], and we strongly believe screening is the first step towards a better disease diagnosis and management. Screening for FGR is a major cornerstone for coordinating care from pregnancy to the postpartum period, which affects both maternal and fetal/neonatal outcomes [86]. The low velocity in which stillbirth and neonatal death rates have decreased in the past 30 years is an “unfinished agenda” [86].

Although the cost-effectiveness of short-term pregnancy-related adverse outcomes is still a matter of debate [87], little is known about the future consequences of a health policy devoted to primary prevention of pregnancy-associated illness in a long-term [49, 59, 88]. On the contrary, the lack of definition of a high-risk group of women that could benefit from a more directed approach delays scientific and clinical evaluation of SGA. As maternal factors have a different magnitude between settings and placental biomarkers are not a reality in most LMIC countries, currently, the 3^rd^ trimester US seems the best approach for SGA prediction [44]. In near future, we envision an integrated approach of pregnant women at booking [85], aiming a transgenerational [49] effect of long-term health, both at individual and populational levels.

## Figures and Tables

**Table 1:** Neonatal adverse events associated with being born SGA.

Perinatal asphyxia
5th-minute Apgar score <7 [10, 11, 14]
5th-minute Apgar score <5 [10, 12, 16, 18]
Admission to neonatal intensive care unit [6, 10, 11, 55]
Hypoglycemia requiring treatment [38, 50]
Phototherapy [50]
Respiratory distress syndrome [12, 14, 16, 50]
Ventilatory support [11, 12, 16, 56]
Necrotizing enterocolitis [12, 56]
Neonatal sepsis [12, 16, 50]
Seizures [12, 16, 18]
Intraventricular hemorrhage [12, 16, 18]
Neonatal death [11, 12, 15, 16]

**Table 2:** Accuracy for clinical factors, ultrasound parameters, and placental biomarkers for SGA prediction (birthweight <10^th^ centile).

Predictive factors	AUC	*S* (95% CI)	Sp (95% CI)	When
Maternal height [64]	0.59	0.43 (0.27–0.60)	0.70 (0.53–0.83)	At booking
Maternal weight [64]	0.57	0.73 (0.60–0.83)	0.35 (0.23–0.51)	At booking
Maternal weight gain [64]	0.60	0.50 (0.42–0.59)	0.66 (0.57–0.73)	At booking
PAPP-A [43]		0.16 (0.14–0.19)	0.90 (0.89–0.90)	1^st^ trimester
PlGF [65]		0.49 (0.44–0.53)	0.64 (0.63–0.66)	2^nd^ trimester
Cerebroplacental ratio^a^ [66]		0.43 (0.39–0.47)	0.94 (0.84–0.98)	3^rd^ trimester
Estimated fetal weight^b^ [67]	0.79	0.38 (0.31–0.46)	0.95 (0.93–0.97)	>32 w
Abdominal circumference [67]	0.92	0.35 (0.20–0.52)	0.97 (0.95–0.98)	>32 w

^a^MCA-PI/UA-PI <10^th^ centile or ≤1.08; ^b^estimated fetal weight <10^th^ centile for gestational age. AUC: area under the receiver operator characteristic curve; *S*: sensitivity; Sp: specificity; PAPP-A: pregnancy-associated plasma protein-A; PlGF: placental growth factor.

## Abbreviations

AC: Abdominal circumference
AFP: Alpha-fetoprotein
AUC: Area under the curve
BW: Birth weight
CPR: Cerebral-placental ratio
DOR: Diagnostic odds ratio
DR: Detection rate
EFW: Estimated fetal weight
FGR: Fetal growth restriction
FL: Femur length
FPR: False positive rate
HC: Head circumference
hPL: Human placental lactogen
HR: Hazard ratio
IUGR: Intrauterine growth restriction
LMIC: Low- and middle-income countries
MCA: Middle cerebral artery
MRI: Magnetic resonance imaging
NT: Nuchal translucency
PAPP-A: Placental protein-A
PlGF: Placental growth factor
POP: Pregnancy outcome prediction
RI: Resistance index
ROC: Receiver operator characteristic
SFH: Symphysial fundal height
SGA: Small for gestational age
UA: Umbilical artery
US: Ultrasound
UtA-PI: Uterine artery pulsatility index
UVBF: Umbilical vein blood flow
VEGF: Vascular growth factor.
